# Can methicillin-resistant *Staphylococcus aureus* prevalence from dairy cows in India act as potential risk for community-associated infections?: A review

**DOI:** 10.14202/vetworld.2017.311-318

**Published:** 2017-03-13

**Authors:** Sathish Gopal, Kurunchi C. Divya

**Affiliations:** 1 Department of Animal Biotechnology, Madras Veterinary College, Chennai, Tamil Nadu, India; 2 Genomics Laboratory. Faculty of Allied Health Sciences, Chettinad Academy of Research and Education, Kelambakkam, Chennai, Tamil Nadu, India

**Keywords:** community associated, dairy cow, livestock associated, methicillin-resistant *Staphylococcus aureus*, methicillin-resistant *Staphylococcus aureus* transmission, milk handlers, monitoring

## Abstract

Methicillin-resistant *Staphylococcus aureus* (MRSA) is classified as hospital associated (HA), community associated (CA), livestock associated (LA) and is a global concern. Developing countries, like India, are densely populated country challenging for public hygiene practices. HA-MRSA is comfortably recorded in India, and CA-MRSA is also reported as increasing one. CA-MRSA is serious disease which affects the community as endemic. MRSA is one among major mastitis-causing organisms in India as LA-MRSA. There were reports for transmission of MRSA as community between milk handlers and cow in global perspective. In India reports of MRSA in short among milk handlers and also transmission between animal and human. Hence, proper monitoring of MRSA transmission in India should be elucidated in account among milk handlers and dairy cows to avoid emerging CA-MRSA as outbreak.

## Introduction

*Staphylococcus aureus* is the well-known epidemic nosocomial pathogen in humans [1,2] and also the primary causative agent of mastitis in cattle [3,4]. *S. aureus* is considered as a significant pathogen with related virulence factors such as slime factor (biofilms), panton-valentine leukocidin (PVL), and some enzymes (proteases, lipases, and elastase), which facilitates destruction of host tissues and metastase to other sites [5], treatment of *S. aureus* infections included semisynthetic penicillin drugs, such as methicillin [6]. However, in the 1960’s, the rise of methicillin-resistant *S. aureus* (MRSA) strains was apparent [7].

MRSA is primarily mediated by the *mecA* gene carried on a mobile genetic element (MGE), the staphylococcal cassette chromosome mec (SCCmec), and at least five types of SCCmec elements have been reported SCCmec type I, II, III, IV, V, VI [8]. To date, SCC elements have been identified within *Staphylococcus sciuri* [9], *Staphylococcus hominis* [10], *Staphylococcus epidermidis* [11], *Staphylococcus haemolyticus* [12], and *S*. *aureus* [13]. *mecA* gene codes for the modified penicillin-binding protein 2a (PBP 2a or PBP 2’). PBP2a is positioned in the bacterial cell wall and has a low binding affinity for β-lactams. The role of inappropriate antibiotic usage, under dosage, and inappropriate administration are also considerable in acquiring antibiotic resistance. MRSA has become apparent as a major cause of hospital-associated (HA) and community-associated (CA) infections [14] and also isolated from milk (livestock associated [LA]) [15].

## HA-MRSA versus CA-MRSA

HA-MRSA (HA-MRSA) characteristically colonizes or infects hospitalized individuals with predisposing risk factors, usually retain SCCmec type I, II or III, and is multi-drug resistant (MDR) [16]. Whereas, CA-MRSA infects healthy individuals without any previous health-care contact, often retains smaller and more mobile SCCmec types, is usually PVL positive, susceptible to non-β-lactam antimicrobial drugs, and frequently evident as skin and soft-tissue infections. However, this difference between CA- and HA-MRSA is gradually dwindling owing to the emergence of *pvl* negative and/or MDR CA-MRSA clones, and its invasion into hospitals. The incidence of HA- and CA-MRSA infections, as well as the relative abundance of different MRSA clones, varies substantially among countries. The HA-MRSA is endemic in many hospitals worldwide [17]. The CA-MRSA has a smaller fitness cost, higher transmissibility and virulence compared to HA-MRSA, and is epidemic in many geographical locations [5]. Limited options are available for the therapeutic management of MRSA infections. The CA-MRSA-associated skin and soft-tissue infections are treated with oral antibiotics including minocycline, doxycycline, clindamycin, rifampicin, sulfamethoxazole, trimethoprim, and fusidic acid. Severe CA-MRSA infections and HA-MRSA requisites intravenous vancomycin therapy. Asymptomatic carriers represent an important MRSA reservoir [18]. In India, INSAR study report [19] showed that the MRSA in 26310 *S. aureus* isolates during the study phase between January 2008 and December 2009 in 15 tertiary centers was 41%. The antibiotic resistant pattern of MRSA isolates in above study indicated that lower susceptibility to ciprofloxacin, gentamicin, cotrimoxazole, erythromycin, and clindamycin, but no isolates found resistant to vancomycin or linezolid.

## Development of CA-MRSA

In recent years, there have been several reports of CA-MRSA infections worldwide; including several outbreaks in the United States [20-22]. Most of these outbreaks have been associated with a single-clone strain. The transmission has occurred by close physical contact in conditions involving children in day-care centers, children, adults, athletes, army personnel, correctional facilities, and homosex [23-25]. Of concern, these patients are otherwise healthy individuals with no known risk factors for MRSA acquisition [26]. In India, the incidence of MRSA shows a large variation, from 6.9% to 81% [27]. As with Gram-negative bacteria in the Indian subcontinent, the widespread use of antibiotics, poor public health infrastructure, and a congested population will probably lead to the emergence and dissemination of antibiotic-resistant lines of *S. aureus*. Additional factors favoring the spread of *S aureus* are high rates of skin infections, such as scabies and impetigo. Recent reports suggest that CA-MRSA is increasing widespread in India with findings from a single-center study in 2011-2012 showing that 65% of CA *S. aureus* infections were due to MRSA, and findings from another study manifests that more than 70% of healthy carriers of *S. aureus* carried MRSA [28]. D’ Souza *et al*. [29] studied the cases of MRSA and found that 54% were true CA-MRSA possessing the *SCCmec IV* and *SCCmec V* genes. These were mainly separated from SSTIs. CA-MRSA isolates also showed variable resistance to ciprofloxacin, erythromycin, clindamycin, and tetracycline. In Brazil, a single nosocomial MRSA clone, the so-called pandemic Brazilian MRSA clone (sequence type [ST]239, SCCmec III), has been responsible for the overwhelming majority of nosocomial infections for the last 20 years [30,31]. Rates of MRSA are up to 60% and are related to an endemic Brazilian clone. A vancomycin-resistant MRSA is also reported in hospital and CA infections of Brazil. Chatterjee *et al*. [32] found the overall prevalence of *S. aureus* nasal colonization was 52.3% and that of MRSA was 3.89% in the community. In major southern districts of Tamil Nadu, 31.1% of staphylococcus strains were found to be methicillin resistant [33].

## Importance of CA-MRSA

Outbreaks of CA-MRSA were first described in the early 1980s and in the 1990s increasing reports began to emerge. CA-MRSA is now a common community-based pathogen demonstrated great geographic diversity with outbreaks reported in the United states, Canada, Europe, Finland, Saudi Arabia, India, Australia, and New Zealand [34]. The emergence and global dissemination of MDR Gram-negative bacteria from India has received much attention. Less attention, however, has been given to records describing the emergence in the last 5 years of two CA-MRSA lines from the India–ST772 and ST22. Both lineages express PVL, which is related to skin and soft-tissue infections. ST772 and ST22 MRSA expressing PVL have become increasingly common in India and have caused frequent outbreaks and infections elsewhere in the world, which is often epidemiologically linked to India [35-37].

## Livestock Associated MRSA (LA-MRSA)

LA-MRSA have emerged in farm animals mainly bovine, swine, companion animals and persons in contact with these animals [38]. Human infections associated with LA-MRSA have also been reported from several parts of the world [39]. Animal to human and *vice versa* of resistant strains can have a potential effect on public health if these strains enter into the community and health-care settings [40]. Transmission of bacterial species between humans and livestock is increasingly being detected in farmers and farm workers in Europe and much of the industrialized world [18]. Despite the fact that *S. aureus* is commonly associated with bovine mastitis, MRSA isolates have been infrequently recorded with the disease. There have been a few reports of MRSA colonization and/or infections in dairy cattle since the very first evidence of MRSA in mastitis in 1972 [41]. Recently, a highly divergent *mecA* gene (now named mecC) in a type XI SCCmec was found in bovine mastitis *S. aureus* [42]. Mastitic MRSA strains from different countries may share similar or different molecular traits. For example, reports from some European countries indicated that ST398 MRSA with SCCmec type IV or V played a vital part in clinical or subclinical bovine mastitis although it was not the only clonal line associated with mastitis [43]. Several genotypes including ST1/t286 MRSA with SCCmec type IVa, ST72/t324 MRSA with SCCmec type IV or IVa, and ST72/untypeable spa-type with SCCmec type IV were reported in Korea [44]. The majority of reported MRSA isolates in Turkey belonged to ST239/spa-type t30 with SCCmec type III, while others belonged to ST8/spa-type t190/SCCmec type IV, orST329/spa-type t30/SCCmec type III [45]. These data indicated that various MRSA clones were associated with bovine mastitis in different parts of the world.

The high incidence of methicillin resistance (47.6%) was recorded from *S. aureus* isolates of dairy farms in China [46]. As India is highest milk producer in the world, dairy production is one of the major agricultural activities among the people. Previous studies from northwest India and Chennai reported the MRSA positive percentages as 13% and 10.94% [47,48]. The higher percentage (19.23%) in this study clearly indicates improper antibiotic usage and poor intramammary administration of mastitis cows. The emergence of MRSA CC398 (known as LA-MRSA) in farm animals and human beings has shown that some *S. aureus* lines might not be strictly host-species restricted. MRSA ST398 can cause infection in people, with close animal contact being the main risk factor, suggesting that farm animals could provide a reservoir of MRSA [49]. Many antibiotic resistance genes are confirmed to have originated in microbes in the environment and have been transferred to other bacteria through MGEs, such as phages [50]. Cui *et al*. [51] reported the presence of MRSA in swine and swine farm workers in four Chinese provinces, all of which belonged to ST9 and spa type t899, possessed a type III SCCmec element and lacked the PVL gene. There was a report on MRSA from pet animals and veterinary staff in China, in which 22 MRSA isolates were distinguished using the API Staph-Ident System, MIC tests, and mecA-specific PCR assay [52]. Another study reported that MRSA of ST97 with SCCmec type IV, ST965 with SCCmec type IV, ST6 with SCCmec type IV, and ST9 with untypeable SCCmec were found in milk samples collected from bovine mastitis cases [53]. Saleha and Zunita [54] discussed the prevalence of MRSA and its impact on public health in Malaysia. They emphasized that there is a need to monitor the presence of MRSA in both pet and domestic animals similar to that in humans to prevent further spread of MRSA. Among 84 staphylococcal isolates were obtained from milk samples from cows, sheep, goats, and buffalo with subclinical mastitis in Rio de Janeiro State of Brazil, no host preference among the animal species have been recorded [55]. MRSA is prevalent in milk from semi-extensive dairy cows in northeastern Brazil, and further investigation on its extent in various types of milk production systems and the farm-to-table continuum is warranted [56].

### Coagulase negative *S. aureus* (CoNS) as mastitic organisms in transmission of methicillin resistance

For instance in Finland, CoNS isolates were predominant from cows with clinical mastitis in which symptoms were most severe in cows with *Staphylococcus hyicus* infection [53]. Of note, human-CoNS species tend to be MDR yet their counterpart, *S. aureus*, is less prone to developing multi-resistance to antimicrobials particularly in the Nordic countries [54]. CoNS species from bovines in Europe are most of the time reported to be susceptible to antimicrobials [57]. Discrepancies in animal husbandry, management practices as well as enforcement of antimicrobial regulations are responsible for this. In veterinary medicine, CoNS have become a problem and are currently incriminated as causes in several recurrences of clinical mastitis.

*S. epidermidis* and *Staphylococcus saprophyticus* are members of CoNS, of that *S. epidermidis* is a common commensal in human skin and also contaminant in implants [58-60]. *S. saprophyticus* is an opportunistic pathogen, causing cystitis and uncomplicated urinary tract infections in women. Coagulase negative *staphylococcus sp*. might be common in subclinical mastitis also [61], but the presence of mecA in these species is a significant one. Jaglic *et al*. [62] reported that mecA was common in *S. epidermidis* (50%). The presence of methicillin resistance in *S. epidermidis* and *S. saprophyticus* implies sequel on environment. Methicillin-resistant *S. saprophyticus* was isolated from dairy products due to environmental contamination [63].

### Transmission of MRSA between human and farm animals (Figure-1)

**Figure-1:**
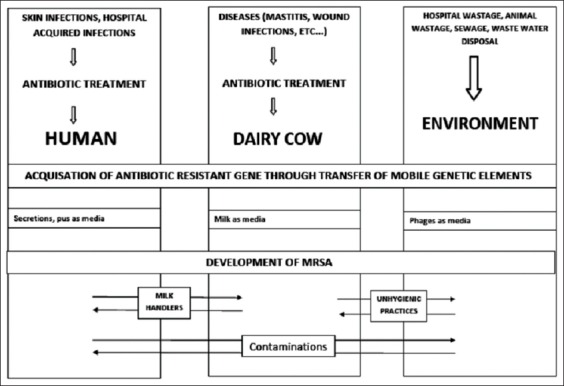
Possibility of methicillin-resistant *Staphylococcus aureus* transmission among human, dairy cow and environment.

Antoci *et al*. [64] evaluated the prevalence and molecular traits of MRSA among dairy farmers in the province of Ragusa, South-Eastern Sicily, their animals and bulk tank milk samples. The results were 36% of human nasal swabs, 61% of bovine nasal swabs, and 44% of bulk tank milk samples. The prevalence of MRSA carrier in humans significantly correlated with the percentage of positive cows on the farm, the number of livestock units, and the presence of positive bulk tank milk samples. Spohr *et al*. [65] found that milk samples of 5.1-16.7% of dairy cows were positive for MRSA; Virgin *et al*. [66] did not identify MRSA from bulk tank milk. Considering that the milking hygiene score correlated with the somatic cell count, which is increased in the presence of mastitis, it is supposable that the improvement of hygiene practices might reduce the risk for MRSA to spread on dairy farms, for example via milkers hands and milking clusters, which represent a common route of transmission for mastitis pathogens, especially *S. aureus* between cows. Prospective studies are needed to investigate MRSA transmission between animals and humans and implement preventive measures. Graveland *et al*. [67] mentioned the possibility of transmission between animals and people who are in close contact with them. A high rate of animal-to-human transmission of CC398 has been reported in pig farming, as well as a significant difference in MRSA prevalence between farmers and their families [68]. Köck *et al*. [69] found that contact with pigs was associated with the risk for MRSA CC398 colonization in a retrospective study among patients admitted to a tertiary-care university hospital. In addition to classical risk factors for MRSA carriage, Harbarth *et al*. [70] suggested to include the evaluation of contact with livestock as an additional risk factor to the admission screening schedule for hospitals, to identify subjects at higher risk for LA-MRSA colonization, who may be responsible for MRSA CC398 introduction in the nosocomial setting and may favor antimicrobial resistance import to hospitals. Prashanth *et al*. [71] assessed the genetic relationship of *S. aureus* isolated from cattle and human in India. In recent times, methicillin-resistant bacteria have been reported in wastewater treatment plants and environmental water samples as well. Since a large part of the antibiotics consumed by humans end up in wastewater, the antibiotics may exert selective pressure resulting in the emergence and transmission of the resistance-conferring genes in antibiotic susceptible organisms; it was proposed [72]. Nonetheless, the presence of β lactamase genes (blaTEM and bla CTX-M9) of *Escherichia coli* and mecA gene of MRSA in bacteriophages DNA isolated from environmental water samples, indicating that phages are reservoirs of resistance genes in the environment, implies that the horizontal gene transfer through MGEs such as plasmids, transposons or bacteriophages might be responsible for the presence of noticeable level of drug resistance in the environment [73-76]. 5 LA-MRSA isolates, 4 of which were obtained from skin and soft tissue infections, were identified from 3687 tested MRSA isolates from persons in Manitoba and Saskatchewan, Canada. Further molecular characterization determined that these isolates all contained SCCmec were negative for PVL and were closely related by macrorestriction analysis with the restriction enzyme Cfr91 [77]. Reports of antibiotic resistance from milk of mastitis-affected dairy farm have been tabulated as Table-1.

**Table-1:** Reports of antibiotic resistance from milk of mastitis affected dairy farm.

References	Country	Disease type	Organism type	Gene type
Honkanen-Buzalski *et al*., [57]	Finland	Clinical mastitis	*Staphylococcus hyicus*	mecA
Kwon *et al*., [78]	Korea	Mastitis	MRSA	SCCmec type IVg
Rabello *et al.*, [79]	Brazil	Mastitis	MRSA	mecA
Hendriksen *et al*., [58]	European countries	Clinical mastitis	CoNS	mecA
Mckay [80]	UK	Unpasteurized milk samples	CoNS	mecA
Huber *et al.*, [41]	Switzerland	Mastitis	MRSA	Type IV a SCCmec - mecA
Fessler *et al*., [59]	Europe	Clinical mastitis	CoNS	mecA
Tu¨rkyilmaz *et al.*, [45]	Turkey	Mastitis	ST239/spa-type t30 ST8/spa-type 190 ST329/spa-type t30	SCCmec type III SCCmec type IV SCCmec type III
Saleha and Zunita [54]	Malaysia	Mastitis	MRSA	Type IV a SCCmec - mecA
Jaglic *et al*., [62]		Clinical mastitis	*Staphylococcus epidermidis*	mecA
Zouhairi *et al*., [63]	lebania	Clinical mastitis	CoNS	mecA
Kumar *et al.*, [47]	India	Mastitis	MRSA	Type IV a SCCmec - mecA
Holmes and Zadoks [43]	European countries	Sub clinical Mastitis	ST398 MRSA	Type IV or Type V SCCmec
Nam *et al*., [44]	Korea	Mastitis	ST1/t286 MRSA ST72/t324MRSA ST72/untypeable spa-type with	SCCmec type IVa, SCCmec type IV or IVa, SCCmec type IV
Garcia-Alvarez *et al*., [49]	UK and Denmark	Mastitis	ST398 MRSA	Type IV or Type V SCCmec
Wang *et al.*, [53]	China	Mastitis	ST97MRSA ST965 MRSA ST6 MRSA ST9 MRSA	SCCmec type IV SCCmec type IV SCCmec type IV Untypeable SCCmec
Pu *et al.*, [46]	China	Mastitis	OS-MRSA	Type IV a SCCmec - mecA
Chandrasekaran *et al.,* [48]	India	Mastitis	MRSA	Type IV a SCCmec - mecA
Paterson *et al.*, [42]	Great Britain	Mastitis	MRSA	Type XI SCCmec - mecC

MRSA=Methicillin-resistant *Staphylococcus aureus*, CoNS=Coagulase negative *Staphylococcus aureus*, SCCmec=Staphylococcal cassette chromosome mec, ST=Sequence type

### Measures to be monitored for avoiding transmission of antibiotic resistant infections

General clean public health, hygiene and sanitary measures should be followed. Development of culturally sensitive awareness campaigns, targeted to the general public, explaining the importance of protecting antibiotics and using them only when absolutely necessary. Provision of education about fundamental hygiene, such as hand-washing, to prevent the spread of infection. It is imperative to improve sanitation systems to eliminate resistant bacteria in wastewater [81].

Some of the WHO guidelines following for human treatment to avoid antibiotic resistance should be considered for animal treatment also. Once the etiology of the infection has been identified on the basis of reliable microbiological methods, antimicrobial therapy is directed at that pathogen. In addition to the constellation of suggestive clinical features, the standard treatment guidelines should include suggestion for diagnostic testing of the disease condition microbiological/pathological, hematological and biochemistry data/values. The recommendation should focus on the investigation for specific pathogens that would significantly alter standard management decisions. Recommendations are generally made for a class of antibiotics rather than a specific drug, unless outcome data clearly favors a specific drug. Since overall efficacy remains good for many classes of agents, the more potent drugs are given preference because of their benefit in decreasing the risk of selection for antibiotic resistance. Other factors for consideration of specific antimicrobials include pharmacokinetics/pharmacodynamics, compliance, safety, and cost. Recommendation on the use of antimicrobials should take into account the use of antimicrobials within the previous 3 months (in which case an alternative from a different class should be selected). In case the individual is from a geographical region that has a high rate (>25%) of resistant organisms reported or where high-level minimal inhibitory concentration is observed then, the use of alternative agents is mandatory [82-84].

Monitoring should be done to ascertain the following:

Is the clinical picture compatible with an infection?
Is there an indication for treatment with antibiotics?
Is the choice of the antimicrobial drug adequate?
  - Broadness of spectrum: Is the spectrum unnecessarily broad? Is the duration of treatment appropriate?
  - Too long/too short? Is the dosage correct?
  - Dose/interval/mode of administration. Is the timing appropriate?
  - Too early/too late?

When treating the animals attending in time, maintenance of treatment, transportation are some of the challenging factors. The veterinarians should have in mind all these while treating the animals with antibiotics.

The human health risks associated with consumption of raw or unpasteurized milk and milk products are well established and have been previously reviewed by Oliver *et al*. [85]. However, the enterotoxin of MRSA is very resistant to heating and pasteurization, boiling of the milk for 1 h leading to a decrease in the quantity of toxin but only autoclaving at 15 psi for 20 min being able to completely destroy the toxin. The sterilized milk needs to be refrigerated at 0°C to 4°C until further processing. Since staphylococci are known to grow well on saline media, the risk for contamination is higher with home-made salted cheeses [86].

### Conclusion

The transmission of MRSA infections may be limited by universal infection-control measures, patient education, screening and decolonization of asymptomatic MRSA carriers in both health-care and community settings. Basic hygiene, good husbandry and biosecurity measures on farms, abattoirs, and food processing units have a tendency to reduce the spread of MRSA in animal population. Individuals with persistent animal contact should be educated on the risk of MRSA transmission in animals or their environment. Global initiatives are urgently needed to monitor the occurrence of and to assess risks posed by emerging clones. For CA-MRSA, emerging evidence suggests that current transmission of particular clones in local community and hospital settings is possible once imported from the Indian subcontinent. Consideration must be given to the screening of patients with a history of overseas travel or health-care contact for both resistant Enterobacteriaceae and Gram-positive bacteria, followed by appropriate infection control procedures. Today, the persistent, indiscriminate, and inappropriate use of antibiotics and the increasing specter of antibiotic resistance are an emerging unfavorable situation for health care. This state needs immediate action with current anti-infective therapies in India. Careful monitoring of the resistance status of *S. aureus* in dairy environments is required, as *S. aureus* transmission is dynamic and involves humans, animals, and likely the farm production environment. Further studies are essential to help identify critical areas that allow for contamination and spread within the farm environment.

### Authors’ Contributions

SG and KCD: Conceived, analysed, drafted and revised the manuscript. Both authors read and approved the final manuscript.
